# Perception and control: individual difference in the sense of agency is associated with learnability in sensorimotor adaptation

**DOI:** 10.1038/s41598-021-99969-4

**Published:** 2021-10-15

**Authors:** Wen Wen, Hikaru Ishii, Ryu Ohata, Atsushi Yamashita, Hajime Asama, Hiroshi Imamizu

**Affiliations:** 1 Research Into Artifacts, Center for Engineering, The University of Tokyo, 7-3-1 Hongo, Bunkyo-ku, Tokyo, 113-8656 Japan; 2 Department of Psychology, The University of Tokyo, 7-3-1 Hongo, Bunkyo-ku, Tokyo, 113-0033 Japan; 3 Cognitive Mechanisms Laboratories, Advanced Telecommunications Research Institute International, 2-2-2 Hikaridai, Seika, Soraku-gun, Kyoto, 619-0288 Japan; 4 Department of Precision Engineering, The University of Tokyo, 7-3-1 Hongo, Bunkyo-ku, Tokyo, 113-8656 Japan

**Keywords:** Psychology, Human behaviour

## Abstract

Adaptive motor learning refers to the ability to adjust to novel disturbances in the environment as a way of minimizing sensorimotor errors. It is known that such processes show large individual differences and are linked to multiple perceptual and cognitive processes. On the other hand, the sense of agency refers to the subjective feeling of control during voluntary motor control. Is the sense of agency just a by-product of the control outcome, or is it actually important for motor control and learning? To answer this question, this study takes an approach based on individual differences to investigate the relationship between the sense of agency and learnability in sensorimotor adaptation. Specifically, we use an adaptive motor learning task to measure individual differences in the efficiency of motor learning. Regarding the sense of agency, we measure the perceptual sensitivity of detecting an increase or a decrease in control when the actual level of control gradually increases or decreases, respectively. The results of structure equation modelling reveal a significant influence of perceptual sensitivity to increased control on motor learning efficiency. On the other hand, the link between perceptual sensitivity to decreased control and motor learning is nonsignificant. The results show that the sense of agency in detecting increased control is associated with the actual ability of sensorimotor adaptation: people who are more sensitive in detecting their control in the environment can also more quickly adjust their behaviors to novel disturbances to acquire better control, compared to people who have a less sensitive sense of agency. Finally, the results also reveal that the processes of increasing control and decreasing control may be partially independent.

## Introduction

The ability to adjust our behavior according to changes in the environment is critical for humans. For example, people can adjust their walking direction and center of gravity to safely cross a stream while sensing the strength of the water’s flow. The processes of adaptive motor learning have been intensively studied in psychology and neuroscience using experimental tasks, such as prism displacement^1, 2^. In such adaptation tasks, sensorimotor feedback is usually perturbed in a novel way, such as an angular displacement between movements and visual feedback. Participants learn to adjust their behaviors to minimize the sensorimotor errors. A previous study revealed large individual differences in adaptive motor learning^3^. In adaptive motor learning, people implicitly or explicitly learn the perturbation in the environment and update their internal model of motor control to generate new motor commands that can minimize prediction errors^4–6^.

On the other hand, when people voluntarily control their body or external tools, a subjective feeling of control, namely the sense of agency in the literature, accompanies the actual control. The sense of agency is considered to be generated from the comparison between predicted sensory feedback and actual sensory feedback^7, 8^. When there is no prediction error (e.g., no mismatch), people feel a strong sense of agency. The sense of agency is closely linked to human consciousness of the self^9, 10^, and it has attracted great attention in various fields such as psychology, neuroscience, psychiatry, and philosophy. However, the current theories of sense of agency mainly consider this subjective feeling as a by-product of successful motor control: people feel a strong sense of agency when they control successfully and lose the sense of agency when they fail to control.

Some recent studies have shown that the sense of agency may also play an important role in motor control by boosting control motivation and facilitating action selection^11, 12^. Such findings reasonably predict that individual differences in the sense of agency affect motor control and learning. To our knowledge, however, no study has directly investigated the influence of the sense of agency on motor control from the perspective of individual differences. The present study probed the relationship between sensitivity in the sense of control and learnability in sensorimotor adaptation. Specifically, the actual level of control was gradually changed in the range of 0–100% using a sensorimotor paradigm^13^. Participants responded as soon as possible when they perceived a change in control (i.e., either an increase or a decrease) from the initial state. The reaction point reflects individual perceptual sensitivity to a change in control. Furthermore, the participants underwent a reaching task that examined their performance in motor adaptation.

Importantly, this study also examined individual differences in the sense of agency when control changes in different directions. Recent studies have shown that perceptual sensitivity is greatly affected by the direction of control change^13–15^, indicating that our cognitive system processes sensorimotor signals in different ways when control increases and decreases. In order to detect the existence of control, people need to explore the environment and find the potential link between sensory feedback and their own actions. Such detection of contingency is known to be developed at a very early stage of development^16, 17^. Adaptive motor learning also requires the ability to detect such contingency and to adjust behavior as a way of minimizing sensorimotor error. On the other hand, in the case of monitoring a decline in control, prediction errors have been considered salient signals^15, 18^. Such detection of sensorimotor error may also trigger adaptation in motor control. In addition, error detection mechanism may be critical for humans to survive in volatile environments, and thus it may be privileged in our cognitive system. However, it is unclear whether the detection of either gain or loss of control is significantly associated with adaptive motor learning. The present study also aims to investigate this question with the approach of considering individual differences, examining if people who are sensitive to the sense of agency also more quickly learn how to control under a sensorimotor disturbance.

In summary, this study examined individual differences in detecting increases or decreases in control, along with the individual differences in a motor adaptation task. The indices of individual perceptual sensitivity of control and adaptive motor learning were pooled to a structure equation model to find the relationship between these indices.

## Methods

### Participants

Sixty healthy adults (mean age: 21.7 years, *SD*: 0.9 years, females: 19) were recruited using a university-wide social media advertisement. All participants had normal or corrected-to-normal visual acuity and were right-handed. We could not conduct a prior power calculation because no previous study could provide an estimated effect size of the correlation between the sense of agency and motor learning. A previous study on the relationship between sensorimotor adaptation and explicit memory reported a correlation of R^2^ = 0.30^19^. Such an effect size requires a sample size of 33 to provide power of 0.95 (α = 0.05, two-tailed test). We decided to use a larger sample than this estimation to ensure sufficient power. All methods were carried out in accordance with relevant guidelines and regulations. The experiments were conducted with the approval of the ethics committee of the Faculty of Engineering at the University of Tokyo. Written informed consent was obtained from all participants before their participation. Participants received a reimbursement for their participation. An exclusion criterion was set before the data collection to exclude participants who excessively responded to no-change trials (see below for details). This criterion excluded six participants, resulting in a sample size of 54 for the analyses.

### Task

The experiment was conducted using two tasks: a control detection task followed by a reaching task. Both tasks were programed using Matlab (R2016b, MathWorks, US) and Psychtoolbox^20, 21^. The visual stimuli were presented on a 22-in LED display (473.76 × 296.10 mm, resolution: 1680 × 1050 pixels, refresh rate: 60 Hz). Participants used their right hand to operate the mouse and their left hand to press response keys on a keyboard.

**Figure 1:**
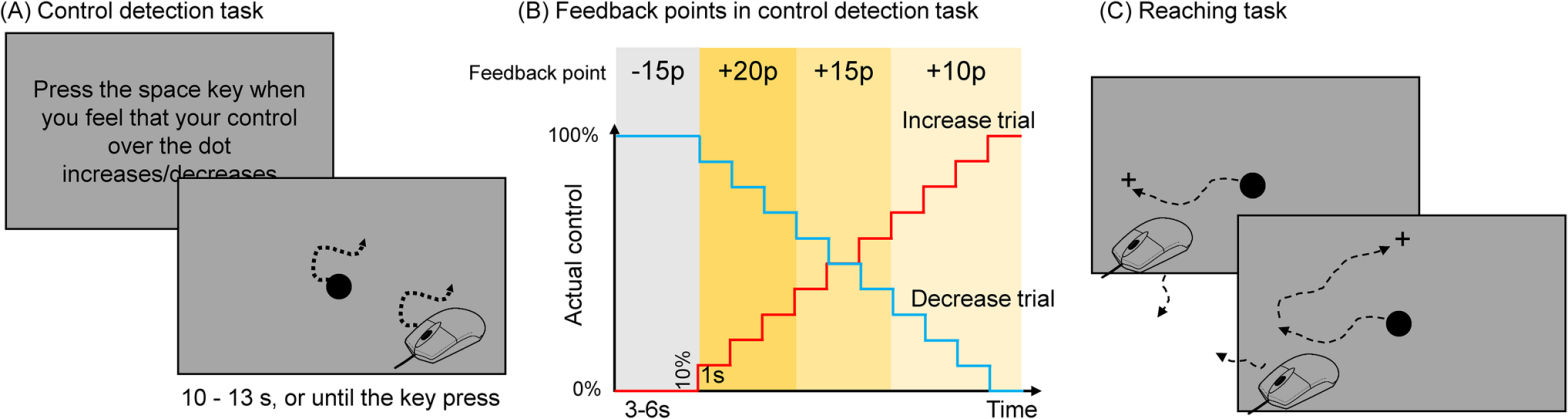
Two experimental tasks. (**A**) Shows an example of the screen in the control detection task, and (**B**) shows the feedback points in that task. (**C**) shows an example of the screen in the reaching task.

Figure 1A shows the timeline of a trial of the control detection task. Increase trials and decrease trials were blocked. The blocked design was used to ensure that participants were able to focus on one direction of change in control in each block. In each trial, participants were told to detect whether their control over a 40-pixel dot (11.28 mm on the screen) increased/decreased or did not change. The instruction of control change direction depended on the block. During each trial, the onset, offset, and speed of the dot always corresponded to the mouse movement. However, the moving direction of the dot was set by an integration of participants’ real-time mouse movements and prerecorded mouse movements^13^. The direction of the mouse movement and the direction of a prerecorded motion were integrated at a certain ratio (e.g., 80/20 in the condition of 80% control) to generate the direction of the visual dot at each refresh frame. The integration ratio thus determines the level of control (0–100%). According to this algorithm, the level of control linearly decreases angular error (i.e., the averaged angular difference between one’s mouse movement and the movement of the visual stimulus)^13^. In an increase trial, participants initially had 0% control over the dot. After a random initial duration between 3 and 6 s, the level of control increased by 10% every 1 s. In a decrease trial, participants initially had 100% control, and their control decreased by 10% every 1 s after a random initial duration between 3 and 6 s. In a no-change trial, participants’ control remained at the initial level (i.e., either 0% or 100%, depending on the type of block) for a random duration between 13 and 16 s. Participants were instructed to pay attention to the motion of the dot and to immediately press the space key on the keyboard when they felt that their control over the dot had changed (i.e., increased or decreased) compared to the beginning of the trial. The dot was replaced by digits indicating feedback points (see below) on the screen after the space key was pressed (Fig. 1B). Participants were told to not press any key if they felt that their control over the dot did not change. If no key was pressed, the increase trial ended 1 s after the control reached 100%. Figure 1B shows the number of feedback points participants received after they pressed the response key. Participants received feedback of + 20, + 15, or + 10 points when they pressed the space key after the control changed and before the extent of change reached 40%, 70%, or 100%, respectively. Participants received no points if they did not respond during a trial. They received − 15 points (i.e., negative feedback) if they pressed the space key before control actually changed or in a no-change trial. The display of the feedback points was designed to encourage participants to respond as soon as they could after feeling a change in control, as well as to discourage them from pressing the response key when there was actually no change in control. There were two blocks: an increase block and a decrease block. Each block contained 20 control-changing (i.e., increase or decrease) trials and 20 no-change trials. The trial order was randomized, and the block order was counter-balanced between participants. Finally, in addition to rewarding points, we also set a criterion to exclude participants who excessively responded to no-change trials. Participants who responded in half or more of the no-change trials in either block were excluded from analyses.

Following the control detection task, participants then performed four trials of a reaching task to examine their motor-learning ability (Fig. 1C). In each trial, participants moved a mouse to control a dot with the goal of touching a cross on the screen. The size of the dot was the same as that of the dot in the control detection task. The size of the cross was 40 pixels (i.e., 11.28 mm), the same as the dot. The dot was initially presented at the center of the screen. At each moment, one cross appeared at one of four possible positions: 394 pixels (i.e., 32.3 mm, 3/8 of the height of the screen) left of, right of, above, or below the center of the screen. After the dot touched the cross, the cross disappeared and then reappeared at a new position randomly chosen from the other three positions. Each trial lasted 200 s. Participants were told to touch the cross with the dot as many times as they could in each trial. There was a visuomotor rotation of either + 90° (i.e., the motion of the dot was rotated 90° clockwise from the motion of the mouse) or − 90° (i.e., the motion of the dot was rotated 90° counter-clockwise from the motion of the mouse) in each trial. This task was designed to measure how fast one could adapt to the visuomotor rotation in each trial. The number of successful touches in each time-window of 20 s was calculated for each trial as the index of motor control performance. We could monitor the change in performance in each trial when participants gradually adapt to the visuomotor rotation. In addition, the four possible positions of the cross also required participants to move the mouse in different directions every time a new cross appeared. This also ensured a certain level of task difficulty to prevent the ceiling effect.

The two types of visuomotor rotation were shifted in an ABAB order and counter-balanced between participants. The ABAB order was designed to ensure that participants had to repeat the adaptation to the visuomotor rotation when a new trial started. It is known that sensorimotor adaptations to the opposite visuomotor rotations interfere with each other, and that the initial adaptation to the bias (e.g., + 90°) is removed by the subsequent adaptation to the other bias (e.g., − 90°)^22–24^. Therefore, in the above ABAB paradigm, we predict that the adaptation in the first trial (A) is removed by the adaptation in the second trial (B). Thus, the initial performance in the third trial (A) reverts to a level comparable to that in the first trial, and participants must re-adapt to the bias in the third trial. In just this manner, the adaptation in the second trial (B) will be removed by the third trial (A). The initial performance in the fourth trial will revert to a level comparable to that in the second trial, which in turn requires re-adaptation by participants. Therefore, we expect participants to show an adaptation or re-adaptation process in each trial of the ABAB paradigm.

### Procedure

Experiments were conducted with participants performing individually in a quiet room. Participants were first introduced to the control detection task, and then they performed four trials for each block (i.e., increase and decrease blocks) as practice. After this practice, participants performed an increase block and a decrease block, each containing 40 trials. After the control detection task, they were then introduced to the reaching task. There was no practice for the reaching task. Participants performed four trials, each lasting 200 s. The experiment took approximately 60 min for each participant.

### Statistical analyses

For the control detection task, we used the level of control measured by the responses in the increase and decrease trials as the indices of perceptual sensitivity to control. The more sensitive one is to a change in control, the sooner they can respond. In addition, we did not use the d-prime of the signal detection theory^25^ as the index of perceptual sensitivity, since the sensory input was impacted by the timing of responses (i.e., the earlier one pressed the response key the less sensory input they received). We first examined the correlation and distribution of the individual response points in control increase and decrease trials.

For the reaching task, we conducted log-linear regressions [Eq. (1)] for each trial of each participant to evaluate the adaptive motor learning progress:

1 $$y=a*\mathrm{log}\left(x\right)+b$$

where *y* represents the number of touches in each time window and *x* represents the number of time windows (i.e., 1–10). The slope *a* reflects the efficiency of sensorimotor adaptation. In other words, *a* reflects how quickly one adapts to the given visuomotor rotation. The intercept *b* reflects the initial motor performance in each trail. We conducted 2 × 2 (visuomotor rotation: first vs second × time of repeat: first vs second) repeated-measures ANOVAs on the slope and intercept to examine the effects of repeat and order of visuomotor rotation on adaptive motor learning.

Finally, and most importantly, we conducted structure equation modelling (SEM) to investigate the relationship between the sense of agency and adaptive motor learning. Perceptual sensitivity to control increase was calculated using the formula of (1 − α); here, α refers to the individual mean control level at which each participant reported perceiving an increase in control. Similarly, perceptual sensitivity to control decrease was the individual mean control level at which participants reported perceiving a decrease in control. For both indices, a larger value was associated with more sensitive perception of control change. Motor learning efficiency was the mean slope of log-linear regression of four trials for each participant. Improvement in initial performance from repeat was the mean difference in the intercept of the log-linear regression between the first and second repeat of the same visuomotor rotation (i.e., trial #1 vs #3 and trial #2 vs #4). Participants were probably able to notice the existence of the repeated visuomotor rotation in trials #3 and #4, and they explicitly recalled the previous trials from memory. Therefore, the improvement in initial performance reflects the memory effect. On the other hand, the slope reflects how fast people can adapt to a visuomotor rotation. These two variables are assumed to reflect different aspects of motor adaptation. The model was designed to investigate the hypothesis that perceptual sensitivity to control (i.e., perceiving control increase and control decrease) influences the individual’s ability of adaptive motor learning. However, SEM cannot exclude the possibility of the reverse causal relationship.

## Results

### Control detection sensitivity

**Figure 2:**
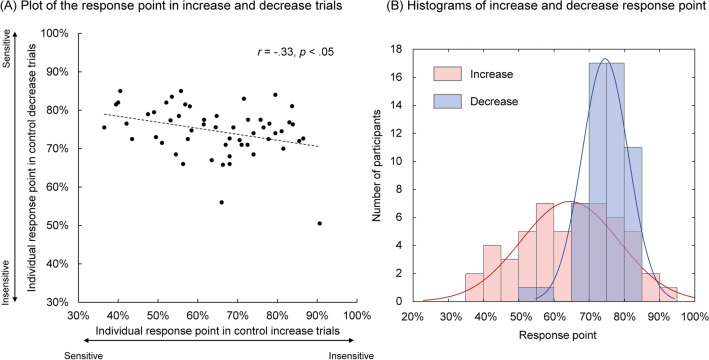
The individual response point in each block (**A**) and the histograms of increase and decrease response points (**B**). The correlation between the perceptual sensitivity to control increase and that to control decrease was significant (*r* = − 0.33, n = 54, *p* < 0.05). The dashed line in (**A**) shows the linear regression line. The solid curves in (**B**) show the fit curves of normal distribution.

The average hit rate and false alarm rate were 97.4% (*SD* = 3.7%) and 18.7% (*SD* = 10.5%) in the increase block and 98.5% (*SD* = 2.7%) and 8.8% (*SD* = 7.5%) in the decrease block. Figure 2A shows the plot of individual response control levels in the control increase and decrease trials. On average, participants reported feeling a change in control after control increased from 0 to 64.6% (*SD* = 13.8%) and after their control decreased from 100 to 74.6% (*SD* = 6.5%). The response control levels were significantly correlated between the increase and decrease conditions (*r* = − 0.33, *p* < 0.05), showing that people who are sensitive in detecting control increase are also sensitive in detecting control decrease. Furthermore, Fig. 2B shows histograms of the response points in the increase and decrease trials. It is worth noting that the individual difference is much larger in the perceptual sensitivity of control increase than control decrease.

### Adaptive motor learning

**Figure 3:**
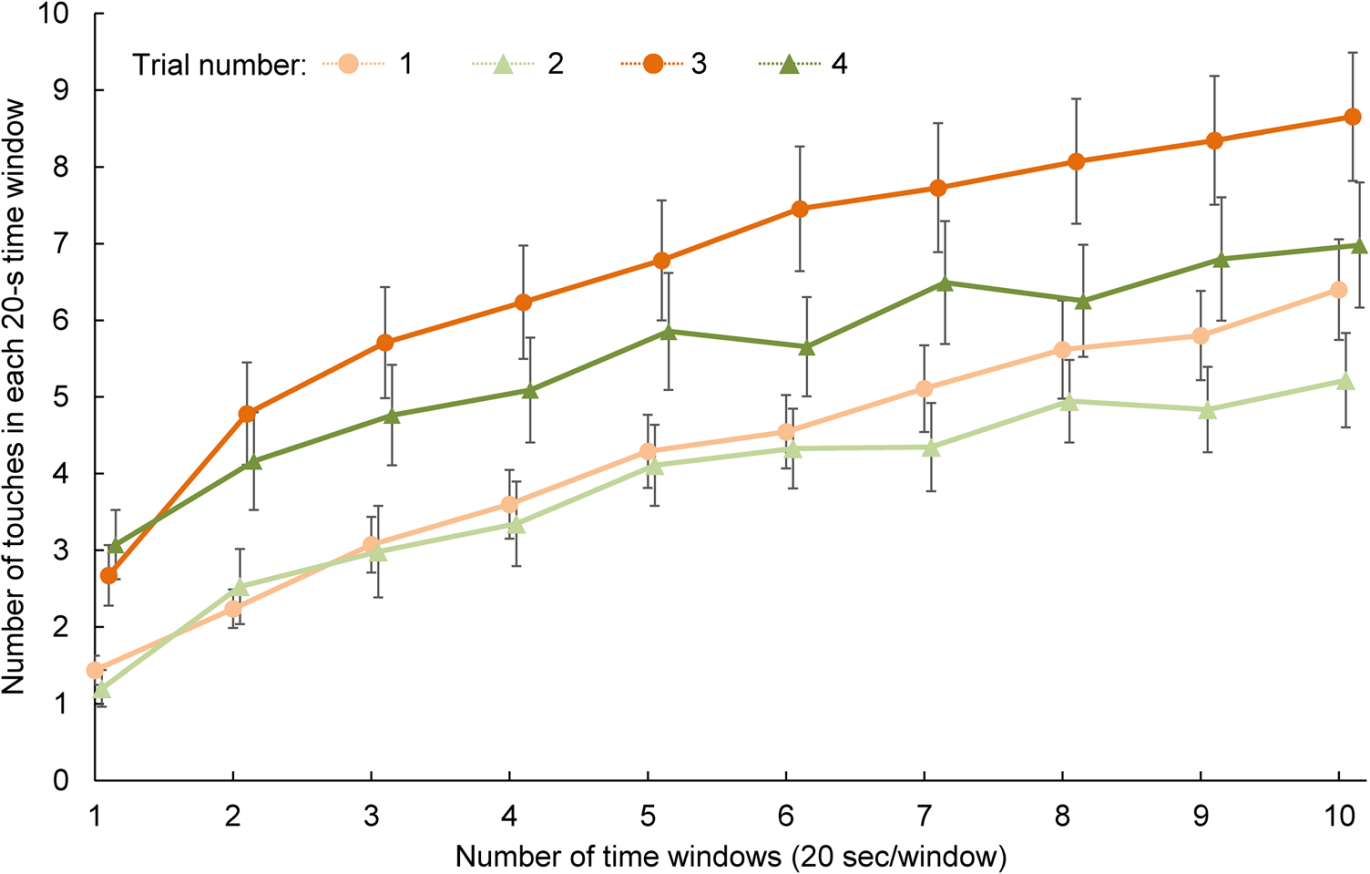
Motor control performance in the reaching task. Error bars represent standard errors.

**Figure 4:**
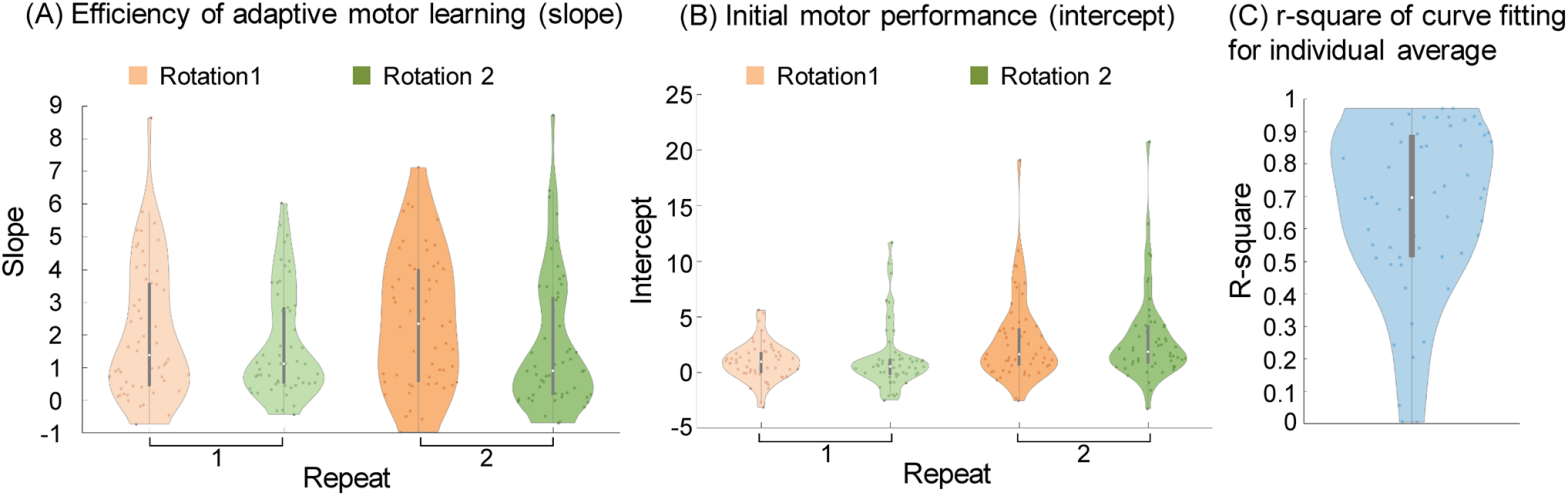
The plots of participants’ slope (**A**) and intercept (**B**) of log-linear regression in each condition; (**C**) the plot of r-square of curve fitting for the individuals’ averages of four trials.

Figure 3 shows the number of touches in each time window (20 s × 10 windows) in each trial. Participants adapted to the visuomotor rotations and gradually improved their performance in each trial. Figure 4A and B show the results of slope *a* and intercept *b* in each condition, respectively. In addition, the overall fitting coefficient (r-square) of each individual is shown in Fig. 4C. As expected, the initial performance was poor in the third and fourth trials, despite the participants having adapted to the same biases in the first and second trails. This poor initial performance was probably caused by the alternating adaptation to the opposite bias (± 90°: see “Methods”). However, the initial performance was slightly better the second time than the first time by about double the number of touches (see numbers of touches for the first time window) when participants had the same type of bias. This better initial performance indicates that the memory of the initial adaptation was partially preserved from the adaptation to the opposite visuomotor rotation. The difference between the first and second time with the same visuomotor rotation (i.e., trial #1 vs #3, trial #2 vs #4) in *b* reflects the preservation of the adaptation memory from the opposite bias.

We conducted 2 × 2 (visuomotor rotation: first vs second × time of repeat: first vs second) repeated-measures ANOVAs on the slope and intercept. Regarding the slope, the main effect of visuomotor rotation, the main effect of repeat, and the interaction between the two factors were all nonsignificant (*F*(1, 53) = 3.55, *p* = 0.065, partial η^2^ = 0.063, *F*(1, 53) = 0.86, *p* = 0.359, partial η^2^ = 0.016, and *F*(1, 53) = 1.57, *p* = 0.216, partial η^2^ = 0.029, respectively). Regarding the intercept, the main effect of repeat was significant (*F*(1, 53) = 25.25, *p* < 0.001, partial η^2^ = 0.323), while the main effect of visuomotor rotation and the interaction were nonsignificant (*F*(1, 53) = 0.323, *p* = 0.572, partial η^2^ = 0.006, and *F*(1, 53) = 0.057, *p* = 0.812, partial η^2^ = 0.001). In summary, the results show that the individual efficiency of adaptive motor learning was constant among trials and that the initial motor performance partially benefited from repetition of the same visuomotor rotation. As an important step, we pooled the above individual indices of control detection sensitivity and adaptive motor learning into a structure equation model to examine the potential relationship between the sense of agency and adaptive motor learning.

### Structure equation modelling

**Figure 5:**
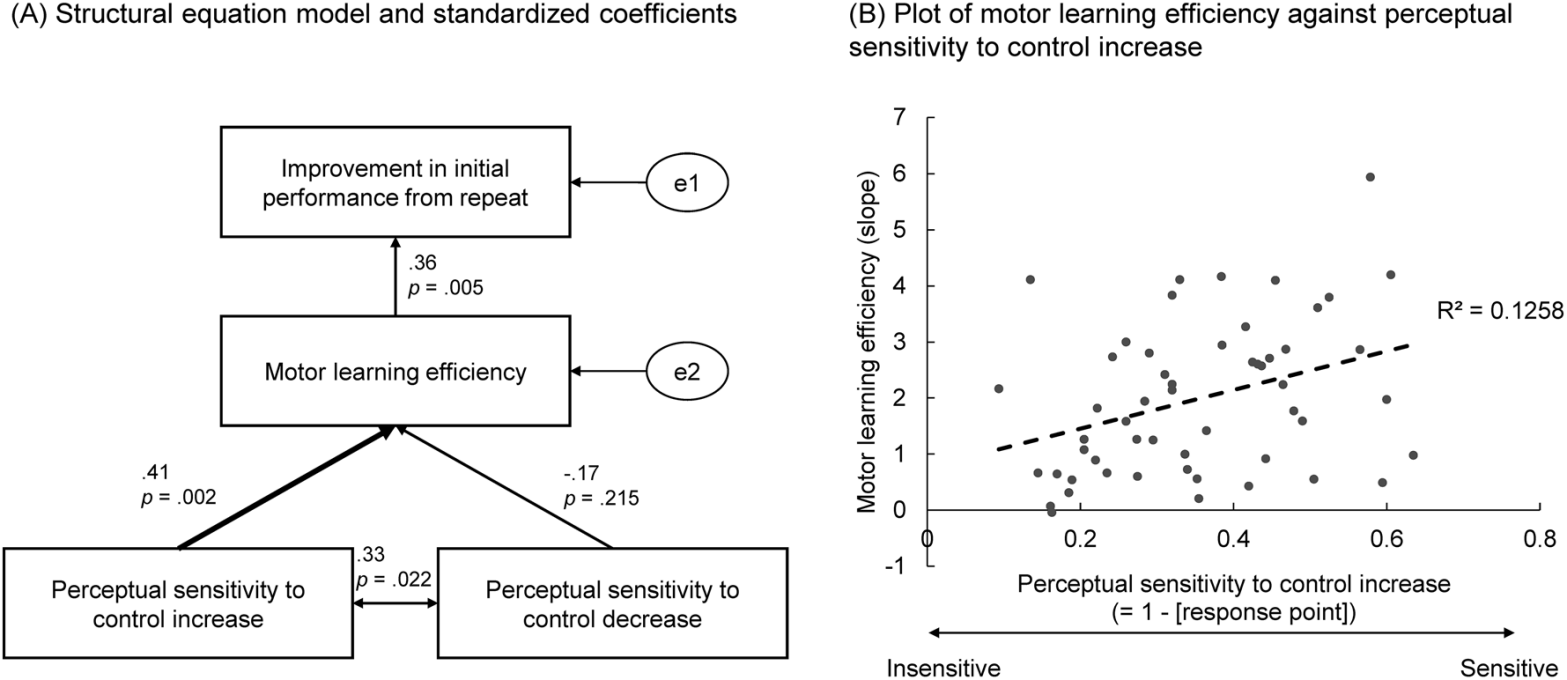
Structural equation model of sense of agency and adaptive motor learning (**A**), and the plot of learning efficiency against perceptual sensitivity of control increase (**B**).

The standardized coefficient of each path of the SEM and the plot of individual motor learning efficiency against perceptual sensitivity to control increase are shown in Fig. 5A and B, respectively. The fit indices are shown in Table 1. The fit indices show that the model well represented the data. The coefficients show that only the perceptual sensitivity to control increase had significant influence on motor learning efficiency, while the influence from the perceptual sensitivity to control decrease on motor learning efficiency did not significantly differ from zero, although the two types of perceptual sensitivity were weakly correlated. In other words, people who are very sensitive when they search for control in a novel environment can also adapt to novel sensorimotor distortion quickly (Fig. 5B). On the other hand, people who are sensitive in monitoring control loss do not show the same superior ability in adaptive motor learning. In addition, motor learning efficiency also significantly influenced the improvement in initial performance when people encountered the same visuomotor rotation for the second time in the ABAB paradigm. This result seems reasonable because the higher learning efficacy permitted a greater number of reaching times in a trial, which might consolidate the adaptation memory against interference from the opposite bias.

**Table 1:** Fit indices of SEM, which show that our model closely fits the data.

	χ^2^/df	GFI	AGFI	RMSEA
Proposed model	0.301	0.994	0.972	0.000
Standard of good fit	< 2.0	> 0.90	> 0.90	< 0.08

## Discussion

The subjective feeling of control, namely the sense of agency, has attracted much attention in cognitive science and cognitive neuroscience due to its close relation to consciousness. Many previous studies have proposed theories and a possible neural basis for the sense of agency. However, its relationship with control—*Is this sense actually important for the action itself?*—remains largely unknown. This study took an approach based on individual differences to resolve this issue. We measured participants’ perceptual sensitivity in detecting a gain of control and in detecting a loss of control, as well as their performance in an adaptive motor learning task. The results from SEM show that the sense of control indeed has a significant impact on the execution of control, but in different ways when people seek control in their environment and when they monitor the decline of control.

Regarding the relationship between the sense and the execution of control, many studies have shown that good execution with satisfying outcomes usually results in a strong sense of agency^26–29^ because people take the outcome of control into account when they judge their own control^30, 31^. However, it has not been clear whether the sense of agency is important for the execution of control. Nor has it even been clear whether the sense of agency can be treated as a type of perceptual or cognitive function, such as visual acuity and working memory capacity. Recently, studies have proposed various types of control detection tasks, in which people search for a visual target under the assumption that control is a potentially useful cue^15^, search for a difference in control among several objects^14, 32^, or judge whether there is a change in control^13^. These tasks are useful for measuring individual differences in the sense of agency. The task in the present study is also in line with the recent assessments of the sense of agency. Building upon those measures of the sense of agency, the results of SEM support our hypothesis that the sense of agency is not only a by-product of the control outcome but also has an impact on how well people adjust their behavior according to external disturbances to achieve better control performance.

Furthermore, an important finding from the present study is that the sense of *finding* control is probably quite different from the sense of *monitoring* the decline of control. Specifically, although the individual perceptual sensitivities to control increase and control decrease correlated with each other, they made different contributions to the individual differences in adaptive motor learning. The sensitivity to control increase significantly contributes to adaptive motor learning efficiency, while the sensitivity to control decrease does not accomplish this. The phenomenon of much larger individual differences in the sense of control increase than in the sense of control decrease is also worth noting. This observation indicates that the processes of sensorimotor signals during control increase and decrease might be partially independent from each other. The detection of control in an ambiguous environment requires explorative behaviors, and the cognitive system might be tuned to search for sensorimotor regularity/contingency^14, 16, 32, 33^. The exploration behavior might show relatively larger individual differences. However, it is unclear whether these large individual differences in finding control are from perceptual sensitivity, which is developed during the span of a lifetime, or from a shorter-span strategy such as attention. It is also unclear whether the sensitivity of finding control can be trained and improved in a short time. On the other hand, when people have already acquired a high level of control in the environment, the monitoring of control decline is similar to an error detection system, which is probably highly prioritized in our cognitive system and shows smaller individual differences. However, this hypothesis of dissociable processes of control increase and decrease has not been directly tested, and thus it is worthy of further investigation through both behavioral and neural approaches.

Finally, basing our approach on individual differences limited our conclusions. It is difficult to make causal inferences based on our results. Further work should consider using experimental manipulation to clarify the true relationship between the perception and execution of control. For example, it would be intriguing to examine whether sensorimotor efficacy improves by training in sensitivity to control increase but not to control decrease. Furthermore, explicit and implicit processes may have very different contributions to adaptative motor learning^34–37^. However, the motor learning in our reaching task did not separate these two types of processes. Although we did not give explicit instruction regarding the visuomotor rotation, the visual feedback at the very beginning of each trial provided cues for the visuomotor rotation. The savings (i.e., improvement at the beginning of the second repeat of the same visuomotor rotation) also indicated explicit learning processes^37^. Further research is required to examine whether the sense of agency is selectively associated with explicit/implicit motor learning. Finally, we used log-linear regression to assess participants’ individual differences in motor learning. However, individual motor performance does not always fit well to log-linear curves (Fig. 4C)^38^. More reliable assessment of individual difference in motor learning is required to further examine the relationship between motor learning and the sense of agency. Nevertheless, the present study shows that the sense of agency is not simply a by-product of control outcome. It is indeed an important *sense*, which is associated with people’s ability to actually exploit their control.

## Data Availability

Original raw data has been deposited to Mendeley Data: Wen, Wen; Ishii, Hikaru; Ohata, Ryu; Yamashita, Atsushi; Asama, Hajime; Imamizu, Hiroshi (2021), “Dataset of study on the individual difference of sense of agency and learnability in sensorimotor adaptation”, Mendeley Data, V3, 10.17632/bbc5ynn2bm.3.
